# *De novo* transcriptome analysis of the Indian squid *Uroteuthis duvaucelii (Orbigny, 1848)* from the Indian Ocean

**DOI:** 10.1038/s41597-024-04112-3

**Published:** 2024-11-16

**Authors:** Nisha Krishnan, Sandhya Sukumaran, V. G. Vysakh, Wilson Sebastian, Anjaly Jose, Neenu Raj, A. Gopalakrishnan

**Affiliations:** 1 ICAR-Central Marine Fisheries Research Institute, Ernakulam North P.O., Kochi, 682018 Kerala India; 2 Mangalore University, Mangalagangotri, Mangaluru, 574199 Karnataka India

**Keywords:** Molecular biology, Transcriptomics

## Abstract

Cephalopods have dominated the oceans for hundreds of millions of years and are unquestionably at the peak of molluscan evolution. The development of the large brain and a well-sophisticated sensory system contributed significantly to its success. Therefore, it is considered the best example of convergent evolution and attracted the attention of scientists from various disciplines of biology. The aim of the present study is to construct a reference transcriptome in the Indian squid *Uroteuthis duvaucelii* to gain insights into cephalopod evolution and enrich the existing cephalopod database. Around 72 million short Illumina reads were generated from five different tissues, including the brain, eye, gill, heart and gonads, and assembled using the Trinity assembler. About 26230 protein-coding sequences were annotated from the assembled transcripts. The BUSCO completeness of the assembly was 71.71% compared to the Mollusca_Odb10 gene set. KEGG and REACTOME pathway analyzes revealed that *U. duvaucelii* shares many genes and pathways with higher vertebrates.

## Background & Summary

Cephalopods are a fascinating and enigmatic group of marine molluscs. They emerged more than 500 million years ago in the late Cambrian^1^ from monoplacophoran like ancestors^2^ and diverged into over 800 living species^3^ with extraordinary traits such as rapid adaptive coloration^3^, ink squirting^4^ and jet propulsion^5^. They have highly diversified body plans and lifestyles compared to other extant animals in the phylum^1,6^. The prehensile cephalic tentacles surrounding the mouth and the muscular funnel derived from the foot are some of the remarkable changes that occurred in the cephalopod lineage from other conchiferans^6^ and the internalization of the shell appears to be a unique evolutionary event in coleoids (squids, octopus and cuttlefish)^7^. In addition, they evolved with large and highly differentiated brains and a sophisticated set of sensory organs with a complex nervous system^8^, which made a greater contribution to the success of their evolution. Hence, they have long been considered the “most advanced” invertebrates. Translocations, exon shuffling, duplications, and gene conversions in the cephalopods could explain the evolution of various morphological and physiological novelties over time^9^. The neuroendocrine system of cephalopods has been found to be similar to that of vertebrates^10^. Together with the presence of a vertebrate-like eye^11^ and intelligence^12^, they offer enormous opportunities to the scientific community for deriving insights into mechanisms of evolutionary convergence, paving the way from physiology to neurobiology to ecology and behavioral science. In fact, cephalopods are the only invertebrates included in the list of regulated species of “Directive 2010/63/EU of the European Parliament and of the Council” on the protection of animals for scientific purposes^13^.

Despite its central role in biological research, genomic and transcriptomic resources are scarce for cephalopods. Recent advancements in next-generation sequencing have enabled genomic sequencing for five species of cephalopods, namely *Octopus minor*^14^, *Euprymna scolopes*^15^, *Octopus bimaculoides*^16^, *Octopus vulgaris*^17^, and *Octopus sinensis*^18^. Obtaining high-quality cephalopod genomes has been complicated due to their large size (e.g., *O. minor* - 5.09 Gb) *O. bimaculoides* −2.7 Gb; *O. sinensis*-2.72 Gb; *O. vulgaris* 2.8 Gb), heterozygosity and high frequency of repeat regions^1,16,19^. The transcriptomic information are available for species such as *Sepiella maindroni*^20^, *O. vulgaris*^21,22^, *Nautilus pompilius*^11^, *Idiosepius paradoxus*^11^, and *Euprymna tasmanica*^23^.

Cephalopods (class Cephalopoda) are represented by an extinct subclass, Ammonoidea and two extant subclasses, Nautiloidea (Nautilus and Allonautilus) and Coleoidea (octopus, squid and cuttlefish)^24^. Among coleoids, squid are not studied as extensively as octopuses, although they have evolved just as much as octopuses and share genetic similarities with other higher vertebrates. In addition, some milestones relate exclusively to squid-related research. Work on the physiology of the giant axon of squid (*Loligo forbesi*) and findings on the excitability of nerve cells led to the Nobel Prize in 1963 for Alan Hodkin and Andrew Huxley^25^.

Among squid, *Uroteuthis duvaucelii* is the dominant species, accounting for about 97% of catches across India each year^26^. It is distributed in the Indo-Pacific at a depth of 30 to 120 m^27^. Commonly known as the Indian squid, it has several characteristics that could make it a valuable model organism for scientific research. It has a relatively short lifespan^28^, which makes it suitable for studying developmental processes, aging, and life cycle dynamics. Due to its abundance and wide distribution, it is amenable to studies on various aspects of its biology. Uroteuthis shares genetic similarities with other cephalopods, which can provide insights into cephalopod biology as a whole. They exhibit complex behaviors such as hunting, mating, communication, and camouflage^29,30^. Studying these behaviors can provide much more valuable insights into neurobiology and behavior. Studying regeneration in *U. duvaucelii*^31^ could provide insights into tissue repair and regeneration in other organisms, including humans. They have a well-developed nervous system, which makes them valuable for neuroscience research and can be used to study neural circuits, learning, memory, and sensory processing. They are valuable species that can be used to understand how environmental changes impact marine organisms and ecosystems because they are sensitive to environmental changes, such as temperature, pH, and pollution^32^. They have unique circulatory and immune systems^23^ that could have implications for biomedical research and serve as a model for studying physiological aspects relevant to human health and disease. Despite these potential advantages, it is worth noting that the use of *Uroteuthis* as a model organism also poses challenges, such as their relatively short lifespan, complex husbandry requirements, and ethical considerations. Nevertheless, with appropriate care and consideration, *Uroteuthis* could be a valuable addition to the list of model organisms used in scientific research.

**Fig. 1:**
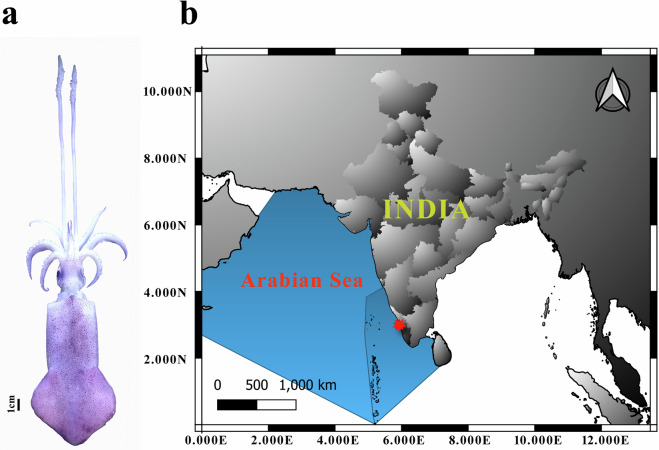
(**a**) *Uroteuthis duvaucelii* (**b**) Sample collection site.

Despite the scientific relevance of squid in all aspects of biology, there is a lack of genomic and transcriptomic information. In the present study, we constructed a reference transcriptome for the Indian squid, *Uroteuthis duvaucelii* (Cephalopoda; Loliginidae) (Fig. 1) based on five tissues including brain, eye, gill, heart and gonad, which can be used as a comprehensive gene catalogue. Assembled contigs were generated from paired end RNA libraries using Illumina Sequencing technology. Approximately 26230 protein-coding sequences were predicted from the assembled transcripts. KEGG and REACTOME pathway analysis and Standalone BlastX against diverse organisms representing different phyla from lower to higher metazoans revealed that *U. duvaucelii* has a high genetic connection with higher vertebrates.

## Materials and Methods

### Ethics statement

No special permits were required for the collection and study of the squid samples from the Arabian Sea (40 m depth) as they come from regular fisheries. The animal experimentation protocols were designed in compliance with the standards of the Institutional Animal Ethics Committee of ICAR-Central Marine Fisheries Research Institute (CMFRI), Kochi. Additionally, the methodologies utilized followed the ARRIVE guidelines (http://arriveguidelines.org).

### Biological samples collection and RNA extraction

Live samples of *U. duvaucelii* were collected using a trawl net operated from research vessel “FV Silver Pompano” at a depth of 40 m from the Arabian Sea (10° 3′53.64″N, 75°55′56.40″E). Species identity was confirmed by both morphological^33^ and molecular analysis comprising DNA Barcoding. Tissues such as heart, gonad, gills, brain, and eye from two male individuals were dissected immediately after collection, snap frozen with liquid nitrogen, and stored at −80 °C until RNA extraction was completed. RNA was isolated following Trizol extraction^34^. Snap frozen *U. duvaucelii* tissues (20–30 mg) was homogenized using an automatic tissue homogenizer (Cole-Parmer 125 Homogenizer) by adding 1 ml of Trizol reagent and incubated for 5 min at room temperature to permit complete dissociation of the nucleoprotein complex. Chloroform (200 μl) (Sigma) was added to the homogenized sample, followed by a brief vortexing and incubation for 2–3 minutes at room temperature. The samples were centrifuged at 12000 rpm for 15 min at 4 °C and the aqueous phase was collected into a fresh tube. To each sample, 500 µl of isopropyl alcohol was added and the RNA was pelleted by centrifugation at 12000 rpm for 10 min at 4 °C. To wash the pellet, 500 μl of 70% ethanol (Fisher Scientific) was used, followed by brief vortexing and centrifugation at 7500 rpm for 5 minutes at 4 °C. The RNA pellet was air-dried for 5–10 min and resuspended in RNase-free water (Qiagen) by passing the solution up and down through the pipette tip several times and stored at −70 °C. All tissue types were pooled using 1 μl of RNA from each of the five tissues to create an RNA library and Illumina sequencing.

### RNA integrity and quality

**Fig. 2:**
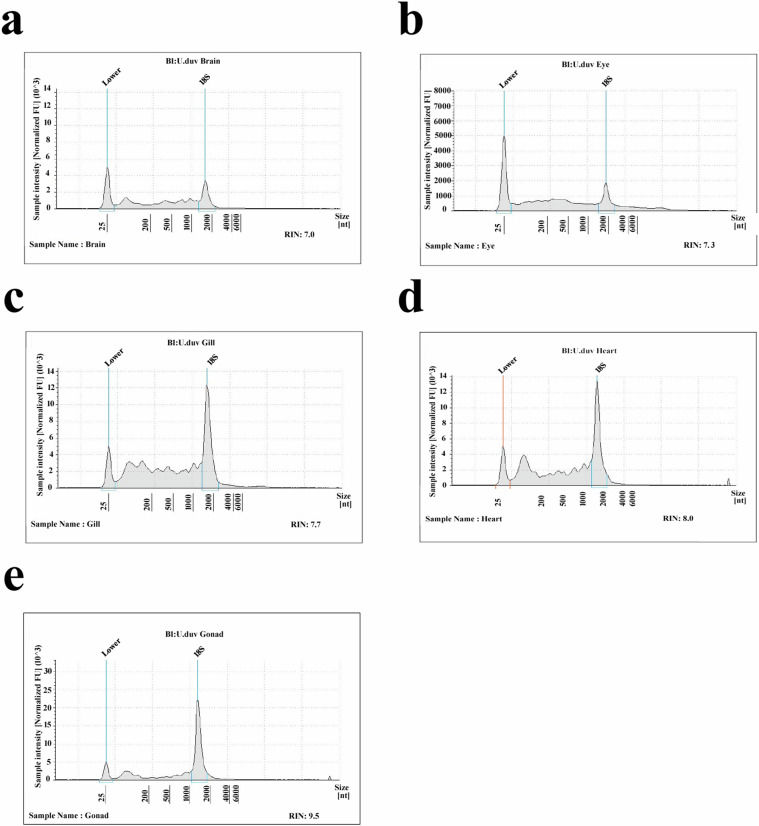
RNA length distribution analysis of (**a**) Brain (**b**) Eye (**c**) Gill (**d**) Heart (**e**) Gonad.

The purity and integrity of the RNA preparations were determined using a Qubit 1.0 Fluorometer (Invitrogen, Carlsad, USA) and a NanoDrop 2000 spectrophotometer (Thermo Scientific). RNA degradation and potential contamination was assayed by agarose gel electrophoresis. RNA integrity was assessed using the Agilent 4150 Tape Station System. Finally samples with an RNA integrity number (RIN) greater than seven (7–9.5) (Fig. 2) and a ratio of 260/280 in the range of 1.72–1.99 were considered for further downstream analysis.

### Library preparation and Illumina sequencing

An mRNA –Seq library was constructed using the TrueSeq RNA Sample Preparation Kit v2 (Illumina, Cat. No. RS-122-2001 and/or RS-122-2002) following the manufacturer’s instructions and sequenced using the Illumina Nanoseq 6000 platform. Poly (A)^+^ mRNA was purified from the total RNA sample of each tissue using oligo dT magnetic beads. The purified mRNA was broken down into short fragments and used as a template for the synthesis of the first strand of cDNA using reverse transcriptase and random hexamer primer. Subsequently, synthesis of the second cDNA strand was performed using RNase H (to remove the mRNA template) and polymerase I (to generate a replacement strand) to form double stranded cDNA. This double-stranded cDNA was end-repaired according to Illumina’s library construction protocol. The end-repaired cDNA fragments were ligated with a PE adapter oligo mix using T4 DNA ligase at room temperature for 15 minutes. The ligated products were purified and separated by size on an agarose gel. DNA fragments of the desired size were excised and sequenced after validation on the Illumina NovaSeq6000 sequencing platform. The total sequencing coverage of the pooled sample was in the order of 72 million reads obtained from both the forward strand (R1) and reverse strand (R2).

### RNA-seq read pre-processing and *de novo* transcriptome assembly

The fastq files of the sequences obtained from the sequencer were pre-processed before performing assembly. During pre-processing, we removed repeated reads, low-quality reads (with a Phred score < 20), and adapter-only reads (less than 13 nucleotides). Additionally, we assessed potential contamination by checking the reads with Kraken 2^35^. We also identified and blasted overrepresented sequences to check for any biological significance before trimming the data using Trimmomatic V0.32^36^. In addition, the rRNAs were removed by aligning with the SILVA database. Overall data quality was assessed using FastQC v0.12.1^37^ for all samples before and after trimming. The resulting high quality reads were then used for *de novo* assembly using the short read assembly program Trinity v2.20^38^ with default settings through OmicsBox version 2.2^39^ platform (https://www.biobam.com/omicsbox/). The assembled transcripts were further clustered using CD-HIT-EST v4.8.1^40^ to reduce redundancy. Clustering was performed using the default parameters of CD-HIT-EST, setting a similarity threshold of 95%. The clustered transcripts were further filtered using TransDecoder 5.5.0^41^ with default parameters, except for retaining ORFs (open reading frames) with a minimum protein length of 100 amino acids. The BLAST step was integrated into the process to improve the accuracy of ORF identification through comparison with known proteins. Simultaneously, the total number of contigs as well as the mean length, N50 length and GC content, were recorded.

### Assessing assembly quality

TransRate^42^ v1.0.3 was used to validate transcriptome assembly. This tool generates various metrics that help identify sources of error in the assembly process and provide evidence regarding the quality of the assembled transcriptome. The quality of the final assembly was assessed through BUSCO (Benchmarking Universal Single-Copy Orthologs)^43^ analysis using OmicsBox platform (version 2.2)^39^ against the Mollusca_Odb10 gene set. It provides quantitative measurements of transcriptome completeness by assessing the presence or absence of conserved orthologs. It assigns a completeness score based on the proportion of expected genes found in the assembly. In addition, missing or fragmented genes can be classified, providing information about possible assembly errors.

### Transcriptome annotation and discovery

**Fig. 3:**
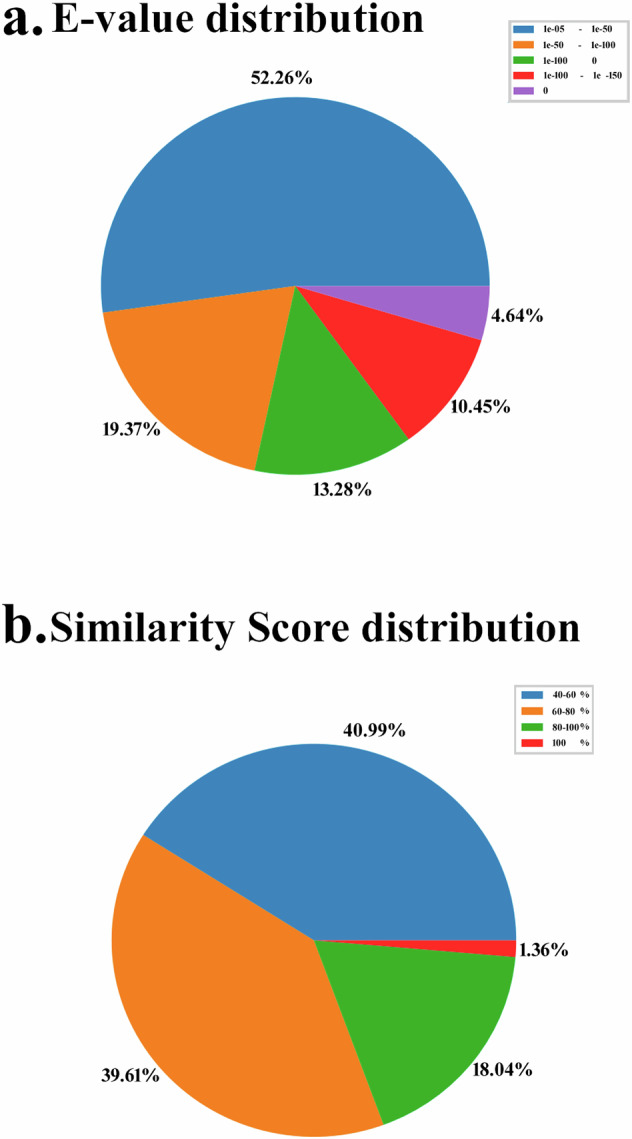
BlastX summary (**a**) E value distribution (**b**) Similarity score distribution of Blast X.

**Fig. 4:**
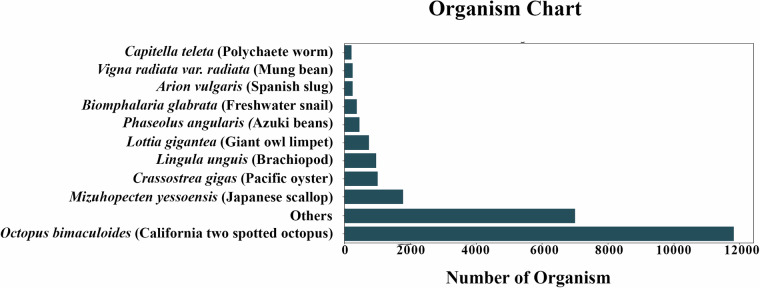
The top 10 BLAST X hits of each transcript after organism annotation.

Annotation was performed on the assembled transcripts/unigenes dataset of *U. duvaucelii* by a Blastx homology search against the UniProt database^44^ and NCBI Nr database, including Mollusca using the BLASTX program (version 2.6.0)^45^ with an E-value cutoff of 10^−3^ to identify putative functional transcripts. The best BLASTX hit was filtered out based on query coverage, identity, similarity score, and description of each transcript. The E-value and similarity score distribution of BLASTX hits are shown in (Fig. 3). In addition, gene function was annotated against GO (Gene Ontology)^46^, PDB (Protein data bank)^47^, KEGG (Kyoto Encyclopedia of Genes and Genomes)^48^, EggNOG (evolutionary genealogy of genes: Non-supervised Orthologous Groups)^49^, InterPro (InterPro protein families and domains database)^50^, RefSeq (Reference Sequence)^51^, OrthoDB (orthologous protein-coding genes Database)^52^, Pfam (Protein family)^53^, PROSITE^54^, BioCyc (Pathway/Genome Databases)^55^, UniPathway^56^, PANTHER (Protein Analysis Through Evolutionary Relationships)^57^, and TIGRFAMs^58^ databases. The top BLASTX hit of each transcript and the organism name were extracted. The top 10 organisms are shown in Fig. 4. The species distribution of the top hits indicates that the majority of *U. duvaucelii* sequences have the highest homology with *Octopus bimaculoides* sequences (11,824 hits, 45.07%).

Functional classification was performed based on Gene Ontology (GO) terms using the Blast2GO^59^ software suite. GO terms were assigned and annotated to transcripts/unigenes according to three main categories mainly biological process (BP), cellular component (CC) and molecular function (MF). The presence of conserved domains in transcripts was analysed using InterProScan^60^ within Blast2GO and the assembled sequences were assigned to the KEGG^48^ and REACTOME pathways^61^.

## Data Records

The COI amplicons, amplified from morphologically identified *U. duvaucelii* specimens, were sequenced using Sanger sequencing and submitted to NCBI, GenBank under the accession numbers PQ163869^62^ and PQ163870^63^. The raw reads from Illumina sequencing and were deposited in the Sequence Read Archive (SRA) of the NCBI database under the Bioproject ID: PRJNA1101856 with the accession number SRR28730639^64^, corresponding to the BioSample ID SAMN40924360. The curated final transcriptome assembly has been submitted to NCBI GenBank under the same Bioproject with accession number GKZI00000000^65^. Additionally, the annotation of *U. duvaucelii* has been deposited in the Figshare database^66^.

## Technical Validation

### Quality of raw reads and Data Filtering

**Fig. 5:**
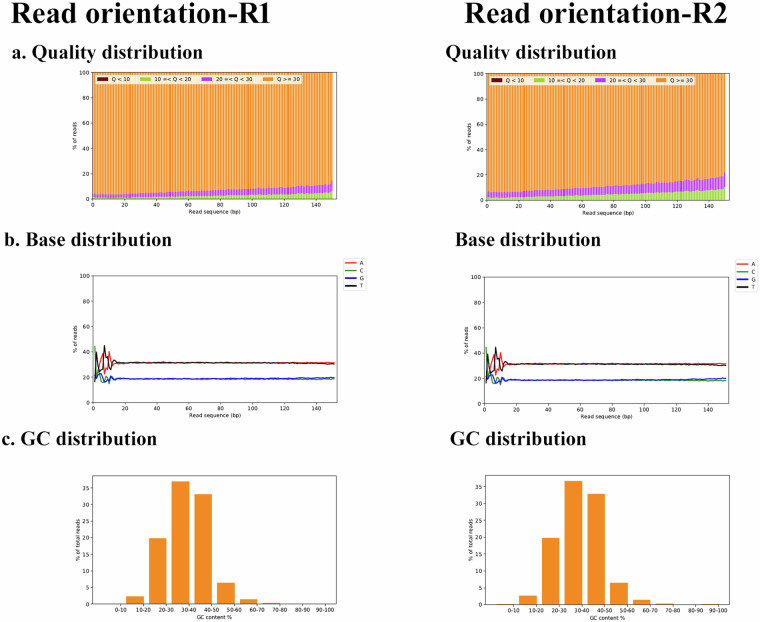
QC report of read orientation R1 and R2. (**a**) Quality distribution (**b**) Base distribution (**c**) GC distribution.

The entire transcriptome profile of *U. duvaucelii* was obtained by paired-end sequencing on the Illumina platform. The number of cleaned reads after trimming is 62504794 (R1 and R2), with a mean read quality (Phred score) 35.75 (R1) and 34.95 (R2) (Table 1). The Q30 percentage (i.e., the percentage of bases with a quality score greater than 30 (Phred score)) is 92.87% for R1 reads, and 88.61% for R2 reads, with corresponding GC contents of 37.61% and 37.64%, respectively (Fig. 5).

**Table 1:** Statistics of cleaned reads (after trimming).

Organism	Sample	Sequencing Platform	Library type	Read orientation	Number of reads obtained	GC%	Number of bases (MB)	Mean read length (bp)	Biosample
*Uroteuthis duvaucelii*	Pooled RNA of Brain, Eye, Gonad, Heart and Gill	Illumina NovaSeq6000	Paired end	R1	31,252,397	37.44	4,166.19	133.31	SAMN40924360
				R2	31,252,397	37.49	3,890.89	124.50

### *De novo* transcriptome assembly and its validation

**Fig. 6:**
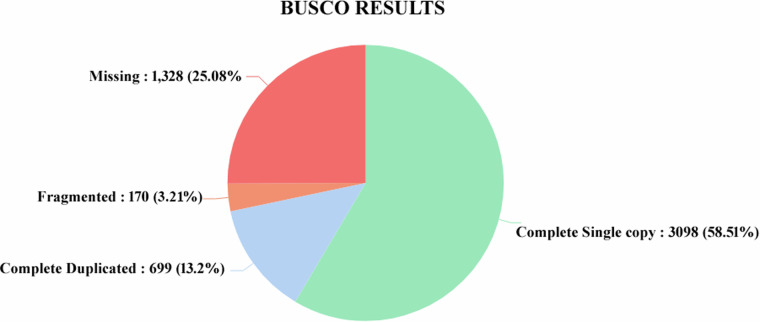
Assembled transcriptome quality. BUSCO score of C:71.71%, F:3.21% and M:25.08%-Mollusca database.

For *U. duvaucelii*, a total of 31,252,397 high-quality reads were obtained after removing adaptor contamination using the Adapter Removal-V2 tool, which were subjected to *de novo* assembly with Trinity, generating 188143 contigs. After CD-HIT clustering and TransDecoder filtering, the assembled transcriptome comprised 88,466 unigenes with a longest transcript length of 24510 bp, a GC percentage of 36.33%, and an N50 of 1359 bp. Transcriptome assembly of *U. duvaucelii* yielded a greater number of unigenes compared to *S. maindroni* (58,224 unigenes)^20^. Validation of our transcriptome assembly was performed using two validator tools: TransRate and BUSCO. The results of the TransRate validation steps are shown in Table 2. In addition, the completeness of the final assembly was assessed by BUSCO analysis on the Mollusca_Odb10 gene set. 71.71% of BUSCO groups have complete gene representation (single-copy and duplicated), while 3.21% are fragmented, and 25.08% are missing. Single-copy BUSCOs and duplicated-copy BUSCOs accounted for 57.8% and 13.2% of complete BUSCOs, respectively. The complete BUSCO scores computed using the Mollusca_Odb10 gene set are shown in Fig. 6.

**Table 2:** Assembly statistics: **Transrate v1.0.3**.

Type	*U. duvaucelii*
Number of Sequences (n_seqs)	88,466
Smallest Contig (bp)	196
Largest Contig (bp)	24,510
Total Number of Bases (n_bases)	74,287,548
Mean Contig Length (mean_len) (bp)	839.73
N50 (bp)	1,359
GC %	36.33
Bases with ‘N’ (bases_n)	4
Proportion of ‘N’ Bases (proportion_n) %	0.00

### Functional Annotation and its quality control

After *de novo* assembly of the transcriptome for the clean reads, all unigenes were applied to gene function annotation and the putative functions were analyzed based on 14 databases. Among the predictable unigenes, a total of 26230 (29.65%, E value-10^−3^) unigenes were annotated with the BlastX alignment against the UniProt database. The E-value distribution of the top hits showed that 47.74% of the sequences have strong homology (less than 1e-60) (Fig. 3). Annotation statistics for other databases such as GO, PDB, KEGG, EggNOG, InterPro, RefSeq, OrthoDB, Pfam, PROSITE, BioCyc, UniPathway, PANTHER, TIGRFAMs are provided in Table 3. Analysis of the distribution of top hit species revealed that the majority of *U. duvaucelii* sequences have the highest homology with the sequences of the cephalopod *Octopus bimaculoides* (11824, 45.07%) (Fig. 4). Furthermore, a sequence similarity search (number of hits) against the genome database of various phyla (Porifera to Chordata) by standalone BlastX is provided in Table 4. While the BlastX results show that the top hits come predominantly from cephalopods, *U. duvaucelii* shares a substantial number of genes with higher vertebrates.

**Table 3:** Annotation statistics.

	Number of Unigenes	Percentage (%)
UniProt	26230	29.65
InterPro	23807	26.91
Pfam	21338	24.12
GO	18283	20.66
Prosite	14855	16.79
RefSeq	11200	12.66
KEGG	8871	10.02
PANTHER	7225	8.16
OrthoB	3350	3.78
TIGRFAM	1835	2.07
EggNog	1373	1.55
Unipathway	342	0.38
PDB	40	0.04
BioCyc	14	0.01

**Table 4:** Standalone BlastX result against various organisms representing different phyla.

Phylum	Class	Organism	Accession Number	Number of Hits
Chordata	Urochordata	*Ciona intestinalis*	GCA_000224145.2	210
Platyhelminthes		*Schistosoma haematobium*	GCA_000699445.3	220
Aschelminthes		*Caenorhabditis elegans*	GCA_000002985.3	254
Porifera		*Amphimedon queenslandica*	GCA_000090795.2	294
Arthropoda		*Drosophila melanogaster*	GCA_000001215.4	393
Cnidaria		*Exaiptasia diaphana*	GCA_001417965.1	613
Mollusca	Gastropoda	*Lottia gigantean*	GCA_000327385.1	1725
Chordata	Cephalochordata	*Branchiostoma belcheri*	GCA_001625305.1	1820
Hemichordata		*Saccoglossus kowalevskii*	GCA_000003605.1	3588
Mollusca	Bivalvia	*Crassostrea gigas*	GCA_902806645.1	3674
Annelida		*Helobdella robusta*	GCA_000326865.1	5490
Echinodermata		*Asterias rubens*	GCA_902459465.3	9628
Mollusca	Cephalopoda	*Octopus bimaculoides*	GCA_001194135.2	77187
		*Octopus vulgaris*	GCA_951406725.2	193824
		*Octopus sinensis*	GCA_006345805.1	193824
Chordata	Vertebrata	*Homo sapiens*	GCA_000001405.29	3312377

### Functional annotation based on GO (Gene ontology) and KEGG pathway analysis

**Fig. 7:**
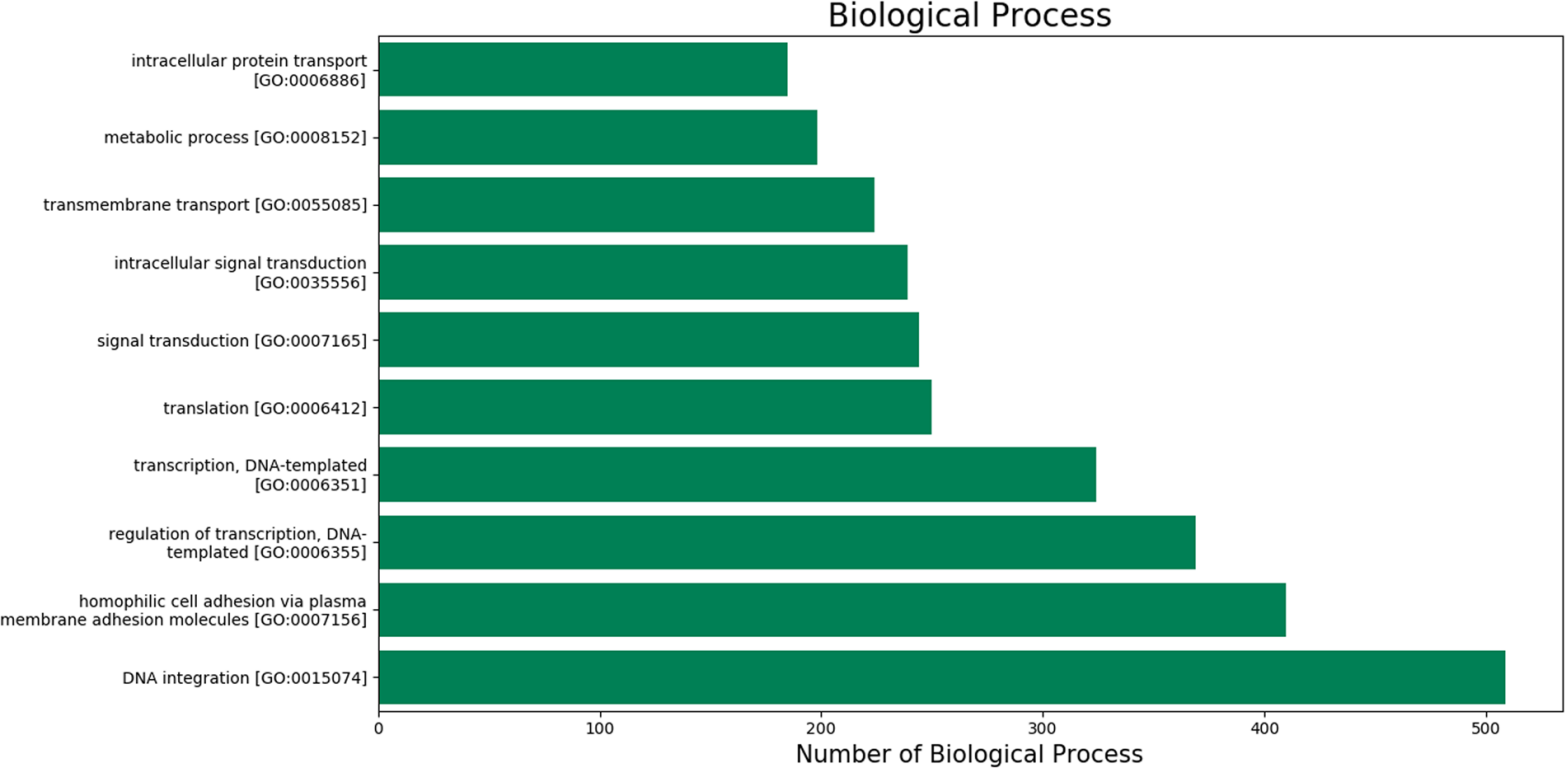
The top 10 GO annotated terms corresponding to ‘Biological Process (BP)’.

**Fig. 8:**
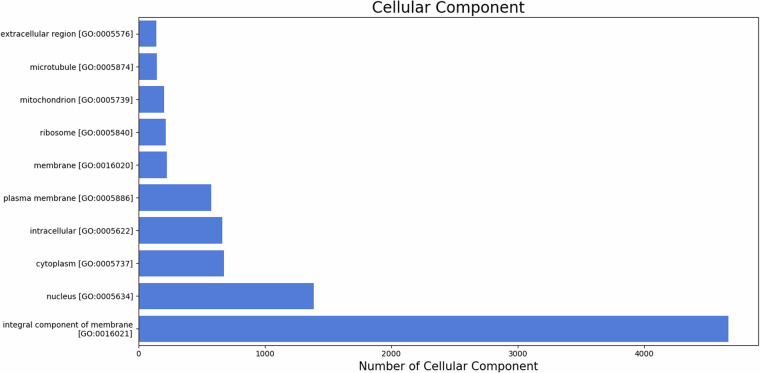
The top 10 GO annotated terms corresponding to ‘Cellular Component (CC)’.

**Fig. 9:**
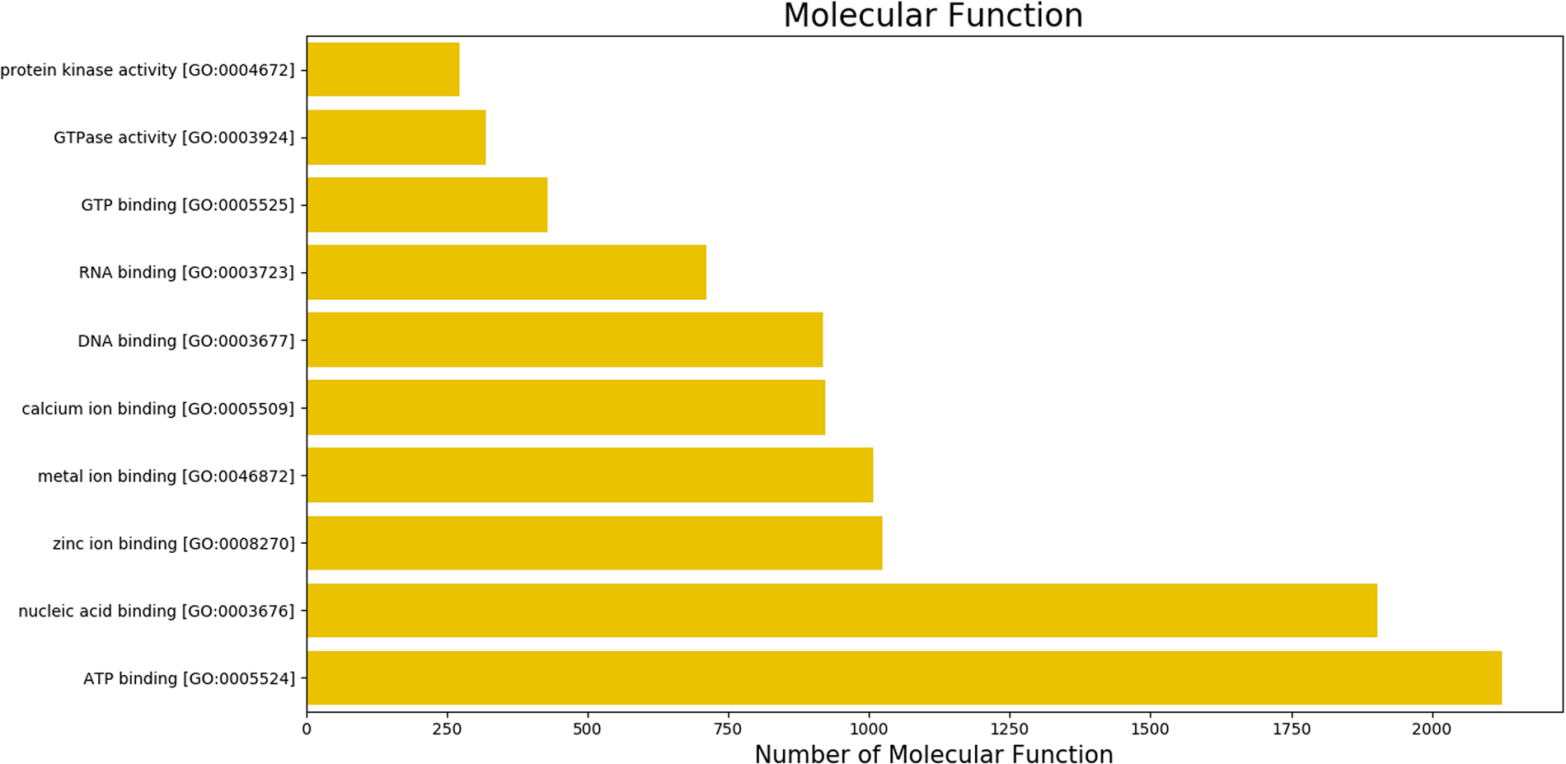
The top 10 GO annotated terms corresponding to ‘Molecular Function (MF)’.

Blast2GO was used to assign GO terms and functionally categorize the assembled *U. duvaucelii* contigs based on gene ontology and orthologous classifications. 10431 unigenes (11.79%) were successfully mapped to existing gene categories, and all unigenes were categorized into 3959 functional groups. The total number of different GO terms identified is 1954 for biological processes, 1428 for molecular functions, and 577 for cellular component categories. The graphical representation of the respective category (number of hits >100) is shown in Figs. 7–9. Among the functional groups, the terms “DNA integration”, “Integral component of membrane” and “ATP binding” were the predominant categories within the biological processes, cellular components and molecular functions, respectively.

To identify the biological pathways active in *U. duvaucelii*, we mapped annotated sequences to reference the canonical pathway in KEGG and REACTOME. A total of 8871 unigenes (10.02%) were assigned to 428 KEGG pathways and 20,015 unigenes (22.62%) were assigned to 10310 REACTOME pathways (Table 5).

**Table 5:** Results of KEGG and REACTOME analyses.

Analysis	Top	pathways	Seq hits >100
KEGG	Metabolism	137	5
	Human disease	96	16
	Organismal system	91	1
	Environmental Information Processing	41	3
	Cellular process	33	4
	Genetic Information Processing	30	1
	**Total**	**428**	**30**
REACTOME	Gene expression (Transcription)	658	7
	Immune System	934	11
	Metabolism of proteins	696	4
	Programmed Cell Death	140	0
	Hemostasis	199	2
	Metabolism of RNA	291	7
	Chromatin organization	60	2
	Developmental Biology	278	0
	Cell Cycle	616	0
	Neuronal System	304	0
	Organelle biogenesis and maintenance	72	0
	Muscle contraction	76	0
	DNA Repair	354	0
	Disease	204	0
	Cellular responses to stimuli	253	0
	Autophagy	61	0
	Cell-Cell communication	53	0
	Circadian Clock	3	0
	Digestion and absorption	23	0
	DNA Replication	129	0
	DNA replication and repair	10	0
	Drosophila signaling pathways	55	0
	Drug ADME	87	
	Extracellular matrix organization	137	0
	Innate Immune System	19	0
	Metabolism	1762	2
	Mycobacterium tuberculosis biological processes	4	0
	Protein localization	46	0
	Reproduction	10	0
	Sensory Perception	54	1
	Signal Transduction	2084	3
	Transport of small molecules	418	0
	Vesicle-mediated transport	220	0
	**Total**	**10310**	**41**

## Data Availability

All data generated or analysed during this study are included in this published article.

## Code availability

No custom code was generated.
